# The role of inflammation and antioxidant defenses in the cardiotoxicity of doxorubicin in elderly CD-1 male mice

**DOI:** 10.1007/s00204-023-03586-1

**Published:** 2023-09-07

**Authors:** Ana Reis-Mendes, Mariana Ferreira, José Alberto Duarte, Margarida Duarte-Araújo, Fernando Remião, Félix Carvalho, Emília Sousa, Maria Lourdes Bastos, Vera Marisa Costa

**Affiliations:** 1 Associate Laboratory i4HB-Institute for Health and Bioeconomy, Laboratory of Toxicology, Department of Biological Sciences, Faculty of Pharmacy, University of Porto, 4050-313 Porto, Portugal; 2 UCIBIO-Applied Molecular Biosciences Unit, REQUIMTE, Laboratory of Toxicology, Department of Biological Sciences, Faculty of Pharmacy, University of Porto, Rua de Jorge Viterbo Ferreira, 228, 4050‐313 Porto, Portugal; 3 Laboratory for Integrative and Translational Research in Population Health (ITR), Faculty of Sport, Research Center in Physical Activity, Health and Leisure (CIAFEL), University of Porto, 4200‐450 Porto, Portugal; 4 1H-TOXRUN–Toxicology Research Unit, University Institute of Health Sciences, CESPU, CRL, 4585-116 Gandra, Portugal; 5 LAQV/REQUIMTE, University of Porto, 4050‐313 Porto, Portugal; 6 Department of Immuno-Physiology and Pharmacology, Institute of Biomedical Sciences Abel Salazar, University of Porto, 4050‐313 Porto, Portugal; 7 Laboratory of Organic and Pharmaceutical Chemistry, Chemistry Department, Faculty of Pharmacy, University of Porto, 4050‐313 Porto, Portugal; 8 CIIMAR–Interdisciplinary Centre of Marine and Environmental Research, 4450‐208 Porto, Portugal

**Keywords:** Cardiotoxicity, Elderly mice, Doxorubicin, Inflammation, Oxidative stress

## Abstract

Doxorubicin (DOX) is a potent chemotherapeutic agent used against several cancer types. However, due to its cardiotoxic adverse effects, the use of this drug may be also life-threatening. Although most cancer patients are elderly, they are poorly represented and evaluated in pre-clinical and clinical studies. Considering this, the present work aims to evaluate inflammation and oxidative stress as the main mechanisms of DOX-induced cardiotoxicity, in an innovative approach using an experimental model constituted of elderly animals treated with a clinically relevant human cumulative dose of DOX. Elderly (18–20 months) CD-1 male mice received biweekly DOX administrations, for 3 weeks, to reach a cumulative dose of 9.0 mg/kg. One week (1W) or two months (2 M) after the last DOX administration, the heart was collected to determine both drug’s short and longer cardiac adverse effects. The obtained results showed that DOX causes cardiac histological damage and fibrosis at both time points. In the 1W-DOX group, the number of nuclear factor kappa B (NF-κB) p65 immunopositive cells increased and a trend toward increased NF-κB p65 expression was seen. An increase of inducible nitric oxide synthase (iNOS) and interleukin (IL)-33 and a trend toward increased IL-6 and B-cell lymphoma-2-associated X (Bax) expression were seen after DOX. In the same group, a decrease in IL-1β, p62, and microtubule-associated protein 1A/1B-light chain 3 (LC3)-I, p38 mitogen-activated protein kinase (MAPK) expression was observed. Contrariwise, the animals sacrificed 2 M after DOX showed a significant increase in glutathione peroxidase 1 and Bax expression with persistent cardiac damage and fibrosis, while carbonylated proteins, erythroid-2-related factor 2 (Nrf2), NF-κB p65, myeloperoxidase, LC3-I, and LC3-II expression decreased. In conclusion, our study demonstrated that in an elderly mouse population, DOX induces cardiac inflammation, autophagy, and apoptosis in the heart in the short term. When kept for a longer period, oxidative-stress-linked pathways remained altered, as well as autophagy markers and tissue damage after DOX treatment, emphasizing the need for continuous post-treatment cardiac monitoring.

**Supplementary Information:**

The online version contains supplementary material available at 10.1007/s00204-023-03586-1.

## Introduction

Doxorubicin (DOX) is a highly efficient chemotherapeutic drug, which is often used in elderly patients (> 65 years) to treat several types of cancer (i.e., leukemia, lymphomas, breast cancer, Kaposi’s sarcoma, soft-tissue carcinomas, gastric, ovarian cancer, liver, and oat-cell carcinoma of the lung). The DOX’s usual dose *per* cycle is 55 to 75 mg/m^2^ every 3 weeks, with a maximal standard cumulative dose of DOX ranging from 400 to 550 mg/m^2^, because of increased risk for cardiotoxicity (McGowan et al. 2017; Reis-Mendes et al. 2015). Heart failure (HF) prevalence was described to be approximately 3% at 400 mg/m^2^, increasing to 7% at 550 mg/m^2^ and to 18% at 700 mg/m^2^ (Cai et al. 2019; Panjrath and Jain 2006). The DOX-induced cardiac dysfunction can manifest acutely during treatment or chronically weeks to years after the end of treatment. Chronic clinical effects may include signs of cardiomyopathy in the form of electrophysiologic changes, a decrease in left ventricular function, arrhythmias, and overt signs of congestive HF. Chronic cardiotoxicity is often irreversible and can be progressive, leading to significant morbidity and mortality (Cai et al. 2019; Panjrath and Jain 2006). Although the incidence of DOX-induced cardiotoxicity is dependent on various factors, including the total cumulative dose of DOX and the dosing regimen, the patient’s age (< 4 years, > 65 years) is considered to play a significant role (Zamorano et al. 2016). Notions on cardio-oncology advocate that pediatric patients may be at increased risk of cardiotoxicity due to the immaturity of the cardiovascular system, while the sensitivity of elderly patients (over 65 years) is due to advanced age and a higher prevalence of pre-existing cardiac conditions (Zamorano et al. 2016). However, pre-clinical data are scarce in these populations.

It is a known fact that oncologists are usually more cautious regarding aggressive therapy in elderly patients, because of preconceived notions of the cardiac fragility of these patients. Although DOX-based chemotherapy usually improves cancer prognosis, physicians are often reluctant to administer this treatment to elderly patients due to the possibility of both short-term and long-term cardiac toxicity (Grann et al. 2006). Hershman et al. showed that advanced age was one of the strongest predictors of withholding DOX treatment and of subsequent congestive HF, and patients older than 80 years had double the risk of developing congestive HF compared to patients ranging from 65 to 70 years old (Hershman et al. 2008). It was accounted that the risk of developing DOX cardiotoxicity increases from 1.6 to 6.8 times in patients older than 65 years (Armenian et al. 2017). Moreover, the elderly population is often overlooked in clinical trials, and due to few studies regarding the real-world elicited cardiotoxicity of chemotherapy, these elderly patients may undergo suboptimal clinical treatment, being more prone to die of cancer.

Several mechanisms have been suggested in DOX-induced cardiomyopathy, including oxidative stress, inflammatory response, mitochondrial dysfunction, autophagy, myocardial fibrosis, Ca^2+^ overload, endoplasmic reticulum stress, and apoptosis (Rocca et al. 2020; Shi et al. 2023). Antioxidant defenses are important for protecting the heart against oxidative damage. As age advances, there may be an increased risk of heart disease due to decreased antioxidant defenses (Lesnefsky et al. 2016). Additionally, age-related changes in the mitochondria include mitochondrial DNA (mtDNA) damage and mitochondrial dysfunction, which can lead to increased reactive oxygen species (ROS) production, further contributing to oxidative stress (Wu et al. 2019a). With advanced age, there is a gradual increase in systemic inflammation, often referred to as inflammaging. Inflammaging is a phenomenon of inflammatory pathogenesis characterized by chronic low-grade inflammation, contributing to higher cardiovascular risk (Franceschi et al. 2018). This inflammation contributes to the decline in cardiac function, and increasing myocardial fibrosis, and size of cardiomyocytes, favoring the development and progression of pre-existent HF (Li et al. 2020; Miyamoto 2019). Moreover, DOX-induced cardiotoxicity has been linked to inflammation (Rocca et al. 2020; Shi et al. 2023), as we have also previously shown (Reis-Mendes et al. 2021b).

The increasing number of cancer survivors, particularly in older ages, and the increase in life expectancy, even after the cancer diagnosis, highlight the need for more research on the long-term safety of cancer drug treatments such as DOX to minimize the risk of adverse events. Therefore, this study aimed to make a broad cardiac evaluation in elderly mice given a clinically relevant cumulative dose of DOX in an approach, as far as we know, used for the first time. The expression of proteins related to oxidative stress, inflammation, and apoptosis in DOX-induced cardiotoxicity in elderly mouse hearts was evaluated, as well as several histological and immunological data gathered after a short period since the last DOX dosing. Moreover, we aimed to go further and assess whether the cardiac damage profile elicited by such DOX cumulative dose persisted or changed 2 months after the clinically relevant cumulative dose of DOX. A more comprehensive understanding of the pathways involved in DOX-inflicted cardiotoxicity, mainly in the long term, would help elucidate whether this elderly population is at higher risk of cardiotoxicity and identify the underlying mechanisms. This knowledge would enable clinicians to make more informed decisions toward more efficacious and safer cancer treatments.

## Experimental procedures

### Materials

Doxorubicin hydrochloride (≥ 98% purity, DOX), Ponceau S, direct red 80, bovine serum albumin, and the all other chemicals used were obtained from Sigma-Aldrich (St. Louis, MO, USA). Phosphate buffered saline solution was purchased from Biochrom (Berlin, Germany), and sodium chloride (NaCl) was acquired from VWR (Leuven, Belgium). Isoflurane (Isoflo^®^) was acquired from Abbott Animal Health (North Chicago, IL, USA). Harris haematoxylin was purchased from Harris Surgipath (Richmond, IL, USA), and 1% aqueous eosin from Australian Biostain (Traralgon, Australia). The Bio-Rad DC protein assay kit was obtained from Bio-Rad Laboratories (Hercules, CA, USA). Primary and secondary antibodies used in Western blot and immunohistochemistry are shown in Supplementary Table S1. Enhanced chemiluminescence (Clarity Western ECL, ref. 1705060) reagents and the Bio-Rad DC protein assay kit were bought from Bio-Rad Laboratories (Hercules, CA, USA). Amersham Protran nitrocellulose blotting membranes (0.45 µm) were supplied by Cytiva (Buckinghamshire, UK).

### Animals

Male CD-1 mice (*Mus musculus*) were acquired as young adults from Charles River Laboratories (L'Arbresle, France) and housed at the Institute of Biomedical Sciences Abel Salazar, University of Porto (ICBAS-UP) rodent animal house facility as described previously (Reis-Mendes et al. 2021a, b). The study was conducted under the license of the local Animal Welfare body (ICBAS-UP process nº. 140/2015) and the General Directory of Veterinary Medicine (process nº. 0421/000/000/2016), in compliance with both Portuguese law (Decreto-Lei nº. 113/2013) and European legislation for the protection of laboratory animals (Directive 2010/63/EU).

### Experimental protocol

Elderly male CD-1 mice aged 18–20 months were used in this protocol. According to the literature, these mice’s age at the beginning of DOX administration corresponds to approximately 78–79 human years old (Wang et al. 2020). All animals received a total of six intraperitoneal (i.p.) injections, administered twice a week, either with a saline solution (NaCl 0.9%, control group) or DOX. In the DOX group, the cumulative dose reached a total of 9.0 mg/kg (DOX being solubilized in sterile NaCl 0.9%). Allometric scaling was employed to ensure that the administered cumulative dose in mice did not surpass the maximum recommended cumulative dose for human DOX therapy (Curry 2010). Therefore, the total dose of 9.0 mg/kg in mice roughly corresponds to 57.0 mg/m^2^ in humans (Beck 2014; Curry 2010; Reagan-Shaw et al. 2008). It is important to note that this dose is significantly lower than the maximum lifelong dose recommended for humans, which ranges from 400 to 550 mg/m^2^ (Reis-Mendes et al. 2015). This cumulative dose allows one to determine early markers of heart damage that allow the study of subtle and even still undisclosed cardiotoxic mechanisms.

The protocol was set as follows, the mice were randomly separated into four groups:

*Two groups for the short-term evaluation of DOX’s cardiotoxicity *(*n* = 10): A group of five animals received a total cumulative dose of 9.0 mg/kg of DOX (1W-DOX group), while another group of five mice received saline treatment (1W-Control group). One week after the final administration, all mice were euthanized for further analysis.

*Two remaining groups for the determination of DOX cardiotoxicity after 2 months *(*n* = 17): A group of nine animals received a total cumulative dose of 9.0 mg/kg of DOX (2M-DOX group), while another group of eight mice received saline treatment (2M-Control group). Animals from these groups were euthanized 2 months after the last administration.

During the experimental period, daily assessments were conducted on food and water intake, body weight, and animal welfare. The criterion for the sacrifice before the end of the protocol was weight loss (more than 10%) or general distress, according to the pain scoring system already described by us previously and approved by the ethical committee (Reis-Mendes et al. 2021a). After the designated drug-free period, the animals were anesthetized with 5% isoflurane and then euthanized by exsanguination. The organs (brain and heart) were removed, weighed, and subsequently used to determine the heart weight to brain weight ratio.

### Histology and immunohistochemistry of the cardiac tissue

The apical segment of heart tissue was fixed in a solution of 4% paraformaldehyde (*w/v*) in PBS at 4 °C and was further dehydrated with grade ethanol solutions, cleared with xylene, embedded in paraffin, and used for histological (haematoxylin and eosin staining and Sirius Red staining) and immunohistochemistry analysis, as described previously (Dores-Sousa et al. 2015; Reis-Mendes et al. 2021a, b). The slides were analyzed in a Carl Zeiss Imager A1 light microscope and images were recorded with a coupled AxioCam MRc 5 digital camera (Oberkochen, Germany). To semi-quantify the severity and incidence of cardiac tissue damage, slides were analyzed for the following parameters: (i) cellular degeneration, (ii) infiltration of interstitial inflammatory cells, (iii) necrotic zones, and (iv) tissue organization, using a scale of 0 to 3, as previously published (Dores-Sousa et al. 2015; Reis-Mendes et al. 2021a, b). The semi-quantitative analysis of immunohistochemistry images and assess collagen deposition were made using ImageJ software (version 1.52a, Wayne Rasband, NIH, Bethesda, MD, USA) and Image-Pro Plus software (version 6, Media Cybernetics, Inc., Rockville, MD, USA), respectively.

### Immunoblotting analysis of the cardiac tissue

One section of the heart was lysed in the complete RIPA lysis buffer and stored at −80 *◦*C for immunoblotting analysis. Immunoblotting analyses were performed according to what has been previously published (Reis-Mendes et al. 2021a, b). The enhanced chemiluminescence ECL reagents were used to detect immunoreactive bands, according to the manufacturer’s instructions. Digital images were acquired using the ChemiDoc Imaging System version 2.3.0.07 (Bio-Rad, Hercules, CA, USA). The images obtained were analyzed using the Image Lab software version 6.0.1 (Bio-Rad, Hercules, CA, USA). Protein content in the whole cardiac homogenate was quantified using the Bio-Rad DC Protein assay. Protein loading of Western blotting was confirmed by Ponceau S staining, as done by us in other works (Reis-Mendes et al. 2021a, b). 

### Statistical analysis

Results are expressed as mean ± standard deviation (SD). Statistical analyses of the animal weight, food, and water intake data were carried out by the two-way analysis of variance (two-way ANOVA) followed by the *Sidak *post hoc test. To assess data normality on assays, the Shapiro–Wilk normality test was performed. When two groups were analyzed, the unpaired *t *test was used when the distribution was normal or by Mann–Whitney test when the distribution was not normal. Statistical significance was considered with *p* values < 0.05. For *p* values < 0.1, a tendency was assumed. To perform the statistical analysis, GraphPad Prism software (version 8.4.2) (San Diego, CA, USA) was used.

## Results

### DOX treatment neither affected body weight nor food and water consumption in aged mice sacrificed at different times

**Fig. 1:**
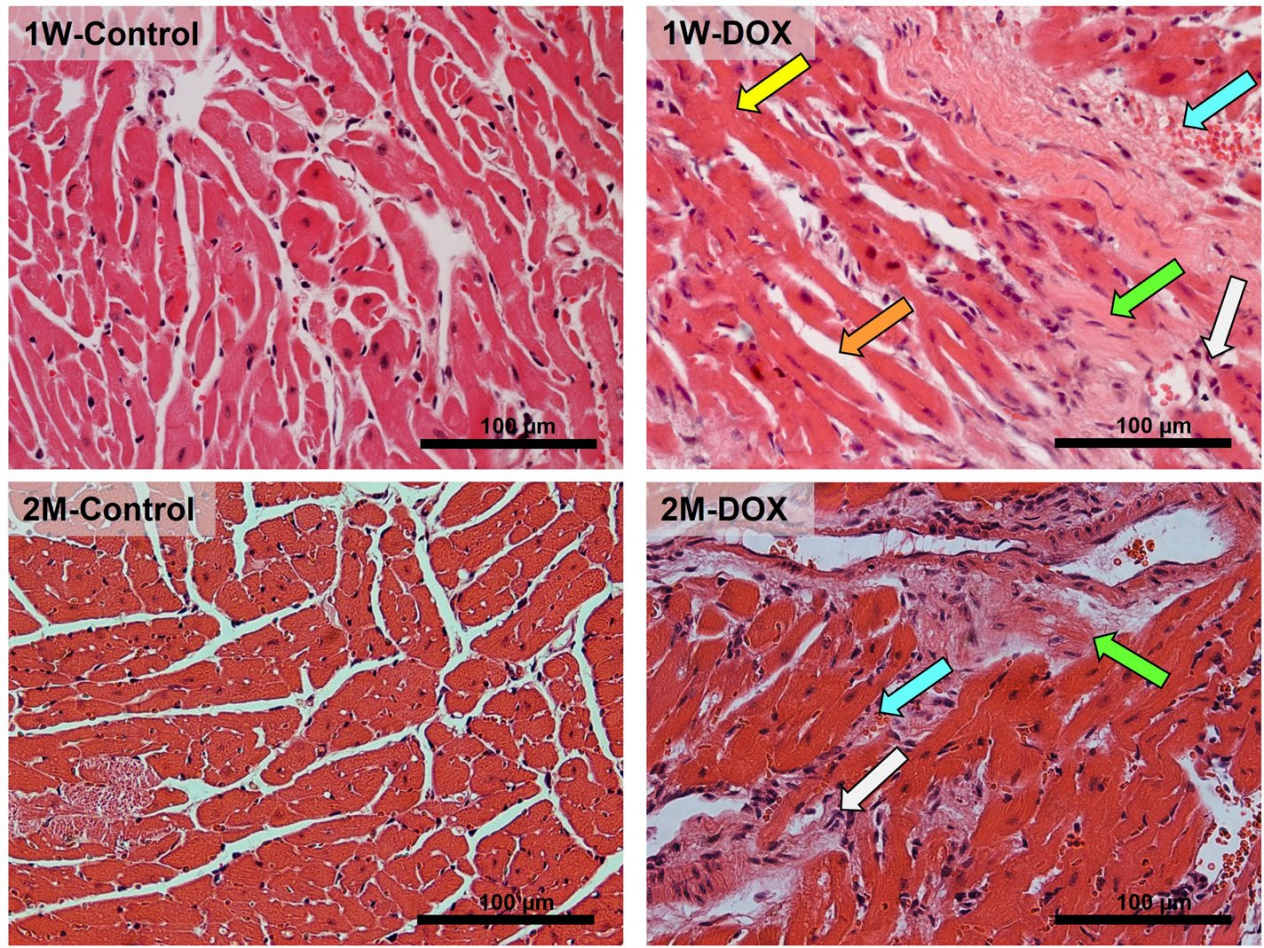
Cardiac histopathology microphotographs of elderly mice, after hematoxylin and eosin staining, sacrificed 1 week after the last administration of DOX (1W-DOX) and control mice (1W-Control); and elderly mice sacrificed 2 months after the last administration of DOX (2M-DOX) and respective control (2M-Control). Representative light micrographs from control mice presented normal morphology and structure, while the DOX-treated mice displayed extensive loss of myofibril (orange arrow), vacuolization (yellow arrow), inflammatory infiltration (white arrow), necrotic zones (green arrow), and vascular congestion (cyan arrow). Scale bar = 100 µm. Representative images of 3 animals per group. Images were taken at 40 × magnification

The 1W-DOX group had no significant differences in body weight, food consumption, and water intake compared to the 1W-Control group (Fig. 1S). In addition, in the 2M-DOX group, body weight, food consumption, and water intake showed no significant differences in comparison to the 2M-Control group (Fig. 1S). The 1W-DOX and 2M-DOX groups did not show any statistically significant differences in heart weight to brain weight ratio compared to the respective control groups (Fig. 1S).

### The 1W-DOX and 2M-DOX groups showed changes in the cardiac structure

Classical hematoxylin and eosin staining was used for histopathological examination of cardiac tissue (Fig. 1). The cardiac tissue from the control groups showed a normal myocardium architecture and a regular cell distribution (1W-Control and 2M-Control). In the 1W-DOX and 2M-DOX groups, ultrastructural alterations, namely dispersed cellular and interstitial edema, diffusion of inflammatory cells, cytoplasmic vacuolization of cardiomyocytes, extensive loss of myofibril, vascular congestion, and necrotic zones were observed (Fig. 1). In both treatment groups, dispersed areas of an intense proliferation of the connective tissue with abundant fibroblast proliferation were observed (Fig. 1).

In the histological assessments, cardiac tissue was characterized by semi-quantitative analysis according to the severity of the following parameters: cell degeneration, interstitial inflammatory cell infiltrate, necrotic zones, and loss of tissue organization (Table 1). In the sections analyzed, the 2M-DOX had less aforementioned damages compared to the 1W-DOX group.

**Table 1:** Semi-quantitative analysis of the morphological parameters (cellular degeneration, necrotic zones, interstitial inflammatory cell infiltration, and loss of tissue organization) in the heart of DOX-treated elderly mice and respective controls

Hematoxylin–eosin staining	1W-control	1W-DOX	2M-control	2M-DOX
Cellular degeneration	0.25 ± 0.25	1.73 ± 0.45****	0.89 ± 0.76	1.48 ± 0.86***
Necrotic zones	0.30 ± 0.25	1.70 ± 0.65****	0.94 ± 0.70	1.68 ± 0.65****
Interstitial inflammatory cell infiltration	0.14 ± 0.26	2.64 ± 0.49****	0.60 ± 0.76	1.38 ± 0.75****
Loss of tissue organization	0.05 ± 0.15	1.82 ± 0.39****	0.42 ± 0.64	1.20 ± 0.90****

Semi-quantitative analysis of the morphological parameters in the heart of elderly DOX-treated mice sacrificed 1 week after the last administration (1W-DOX) and control mice (1W-Control), and DOX-treated mice sacrificed 2 months after the last administration (2M-DOX) and respective controls (2M-Control). Results, given in scores, are presented as means ± standard deviation (SD) and were obtained from 3 animals of each treatment group (ten random fields *per *animal). Statistical comparisons were made using the Mann-Whitney test between the groups: ***p < 0.001, ****p < 0.0001, DOX *vs.* control.

### The 1W-DOX and 2M-DOX groups showed myocardial fibrosis

**Fig. 2:**
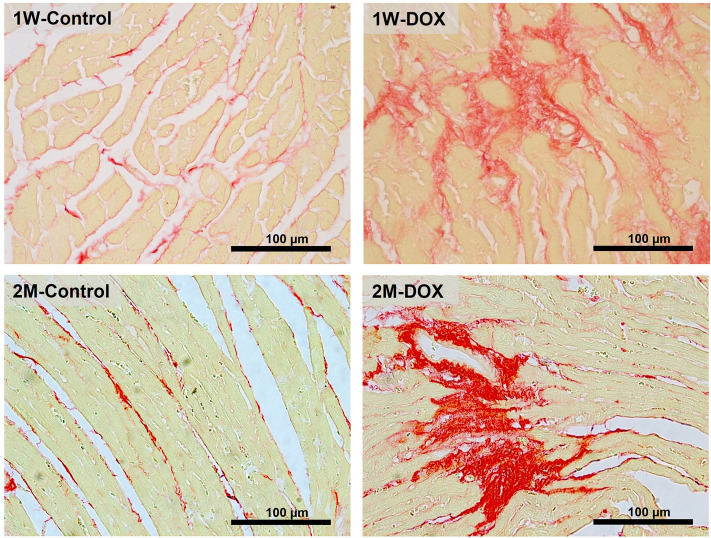
Sirius red staining assessed by light microscopy of fibrosis in the heart of DOX-treated elderly mice sacrificed 1 week after the last administration (1W-DOX) and control mice (1W-Control), and DOX-treated mice sacrificed 2 months after the last administration (2M-DOX) and respective controls (2M-Control). Scale bar = 100 µm. Representative images of 3 animals per group. Images were taken at 40 × magnification

Sirius Red was used for collagen deposition evaluation in the cardiac tissue (Fig. 2). The 1W-DOX and 2M-DOX had a significant increase in collagen deposition in the cardiac tissue (Table 2) consistent with interstitial cardiac fibrosis in comparison with the respective control group (Fig. 2). The increase in fibrous tissue in the heart occurred in the two groups administered with DOX, compared with the respective controls.

**Table 2:** Semi-quantitative analysis of fibrosis in the heart of DOX-treated elderly mice and respective control

	1W-control	1W-DOX	2M-control	2M-DOX
% of collagen *per* total section area	8.40 ± 2.27	28.18 ± 17.42*	3.18 ± 1.12	22.63 ± 10.80**

Collagen content red staining of DOX-treated elderly mice sacrificed 1 week after the last administration (1W-DOX) and control mice (1W-Control), and DOX-treated mice sacrificed 2 months after the last administration (2M-DOX) and respective controls (2M-Control). Results are expressed as a percentage of collagen per total section area, and presented as means ± SD and were obtained from 3 animals of each treatment group (six random fields per *n*). Statistical comparisons were made using the Mann-Whitney test between the groups: *p < 0.05, **p < 0.01, DOX *vs.* control.

### The 1W-DOX group showed a significant increase in iNOS expression while the 2M-DOX group showed a significant increase in glutathione peroxidase expression

**Fig. 3:**
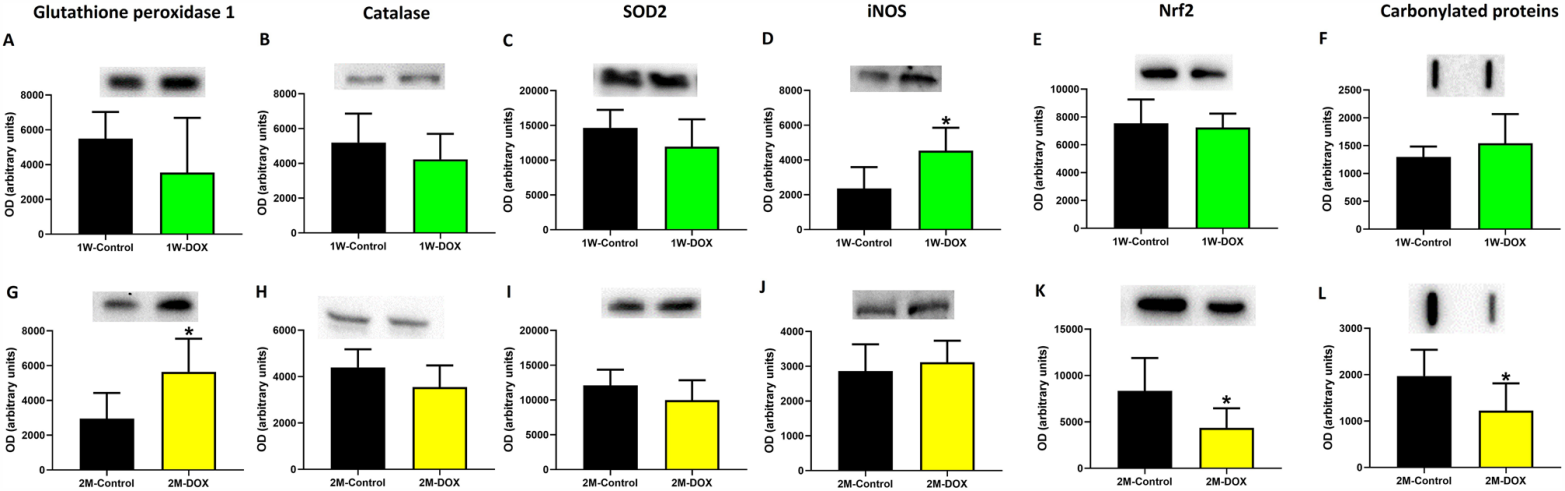
**A**, **G** Glutathione peroxidase (22 kDa), **B**, **H** catalase (60 kDa), **C**, **I** superoxide dismutase 2 (SOD2) (26.6 kDa) and **D**, **J** inducible nitric oxide synthase (iNOS) (131 kDa), **E**, **K** nuclear factor erythroid-2-related factor 2 (Nrf2) (97 kDa) expression in the cardiac tissue were evaluated by Western blotting. **F**, **L** Protein carbonylation cardiac content was evaluated by slot blot. **A**–**F** elderly mice sacrificed 1 week after the last administration of DOX (1W-DOX) and control mice (1W-Control); and **G–L** elderly mice sacrificed 2 months after the last administration of DOX (2M-DOX) and respective controls (2M-Control). Values are expressed as mean ± SD and were obtained from 4–5 (1W) or 5–9 (2M) animals from each treatment group. Statistical comparisons were made using the unpaired *t *test: *p < 0.05, DOX *vs*. control. OD: optic density. Protein loading was confirmed by the Ponceau S staining (Figure S2)

In the 1W-DOX group, iNOS expression increased significantly when compared with the 1W-Control group (Fig. 3D). No other significant changes were reported in 1W-DOX namely on glutathione peroxidase, catalase, superoxide dismutase (SOD2), Nrf2, and carbonylated proteins expression (Fig. 3A, B, C, E, F).

The 2M-DOX group showed no significant differences in catalase, SOD2, or iNOS expression after DOX administration in heart tissue (Fig. 3H–J). The 2M-DOX group showed an increase in glutathione peroxidase (Fig. 3G) and a decrease in Nrf2 and carbonylated protein expression (Fig. 3K, L) in comparison with the respective control group.

### The 1W-DOX group showed increased IL-33 and decreased IL-1β expression

**Fig. 4:**
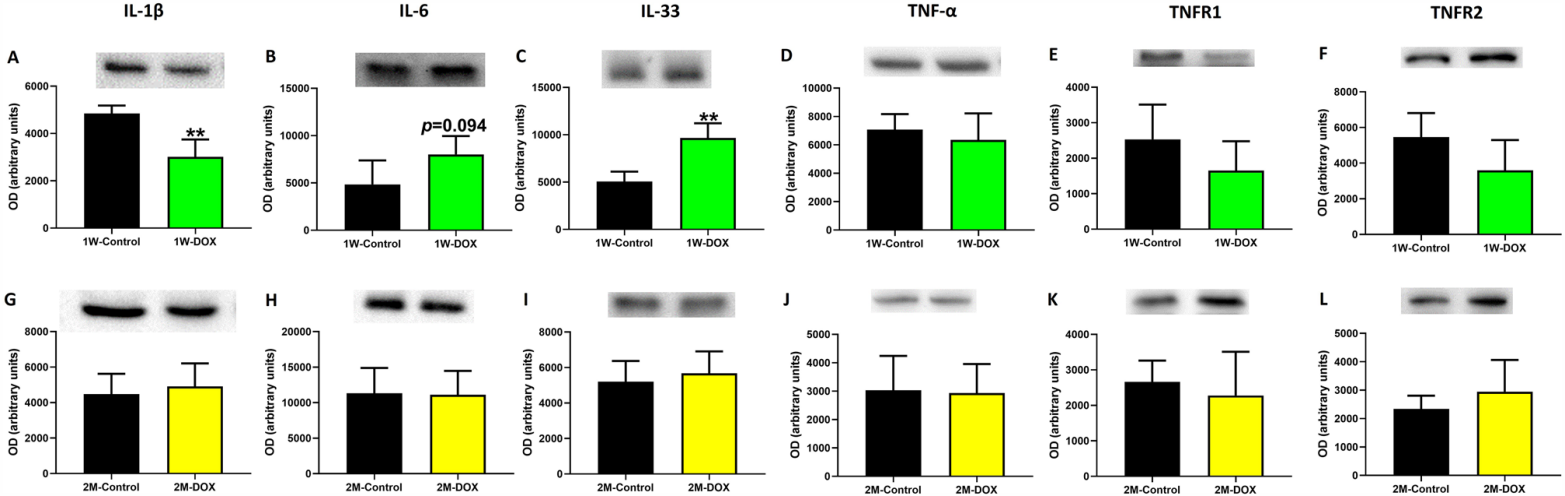
**A**, **G** Interleukin-1β (IL-1β) (35 kDa), **B**, **H** interleukin-6 (IL-6) (23 kDa), **C**, **I** Interleukin-33 (IL-33) (33 kDa), **D**, **J** tumor necrosis factor- α (TNF-α) (25 kDa), **E**, **K** type 1 TNF receptor (TNFR1) (50 kDa) and **F, L** type 2 TNF receptor (TNFR2) (75 kDa) expression in the cardiac tissue evaluated by Western blotting, in **A–C** elderly mice sacrificed 1 week after the last administration of DOX (1W-DOX) and control mice (1W-Control); and **D–F** in elderly mice sacrificed 2 months after the last administration of DOX (2M-DOX) and respective controls (2M-Control). Values are expressed as mean ± SD and were obtained from 4–5 (1W) or 5–6 (2M) animals from each treatment group. Statistical comparisons were made using the unpaired *t *test: **p < 0.01, DOX *vs*. control. OD: optic density. Protein loading was confirmed by the Ponceau S staining (Figure S3)

The 1W-DOX group showed a decrease in IL-1β expression (Fig. 4A), a significant increase in IL-33, and a trend toward an increase in IL-6 (*p* = 0.094) expression as compared to the 1W-Control group (Fig. 4B, C). At 1W, no changes in tumor necrosis factor-α (TNF-α), type 1 tumor necrosis factor receptor (TNFR1), and type 2 tumor necrosis factor receptor (TNFR2) expression were seen (Fig. 4D–F). No significant differences in IL-1β, IL-6, IL-33, TNF-α, TNFR1, and TNFR2 expression were seen in the 2M-DOX group when compared to the 2M-control group (Fig. 4G–L).

### The 1W-DOX group showed a significant decrease in p38 MAPK expression in the cardiac tissue

**Fig. 5:**
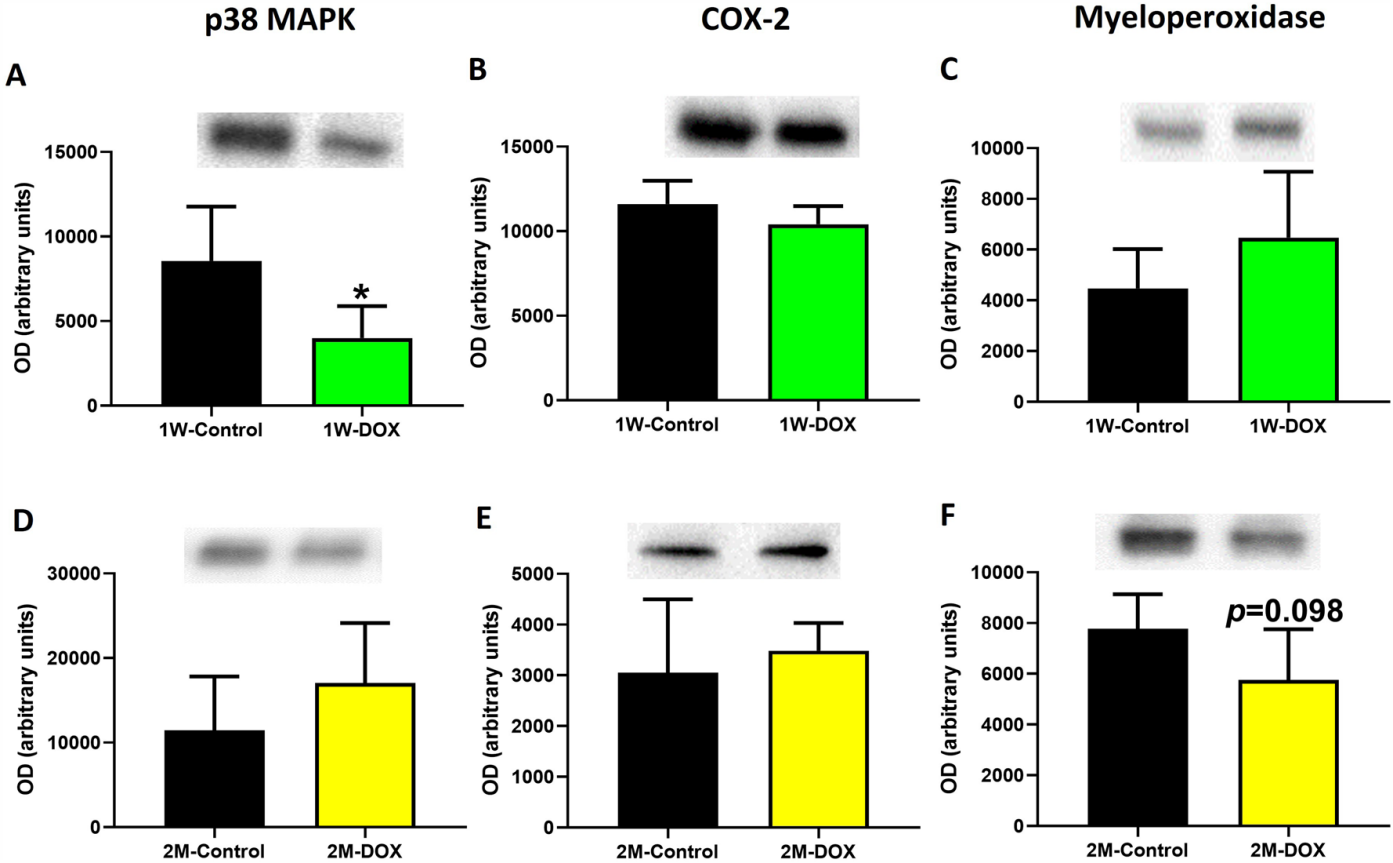
**A**, **D** p38 mitogen-activated protein kinase (MAPK) (40 kDa), **B**, **E** cyclooxygenase-2 (COX-2) (75 kDa), **C**, **F** myeloperoxidase (63 kDa) expression in the cardiac tissue evaluated by Western blotting, in **A, B, C** elderly mice sacrificed 1 week after the last administration of DOX (1W-DOX) and control mice (1W-Control); **D–F** elderly mice sacrificed 2 months after the last administration of DOX (2M-DOX) and respective controls (2M-Control). Values are expressed as mean ± SD and were obtained from 4–5 (1W) or 5–6 (2M) animals from each treatment group. Statistical comparisons were made using the unpaired *t *test: *p < 0.05, DOX *vs*. control. OD: optic density. Protein loading was confirmed by the Ponceau S staining (Figure S4)

The 1W-DOX group had a decrease in p38 MAPK expression compared to the 1W-Control group (Fig. 5A). In the 1W-DOX group, no changes in cyclooxygenase-2 (COX-2) and myeloperoxidase expression, compared to the 1W-Control group, were observed in the heart (Fig. 5B, C). The 2M-DOX group showed a trend toward decreased myeloperoxidase (*p* = 0.098) expression in comparison to the 2M-Control group (Fig. 5F). In the 2M-DOX group, no significant changes were observed in p38 MAPK and COX-2 expression when compared to the 2M-Control group (Fig. 5D, E).

### The 2M-DOX group showed a significant decrease in the nuclear factor-ĸB (NF-κB) p65 in cardiac tissue

**Fig. 6:**
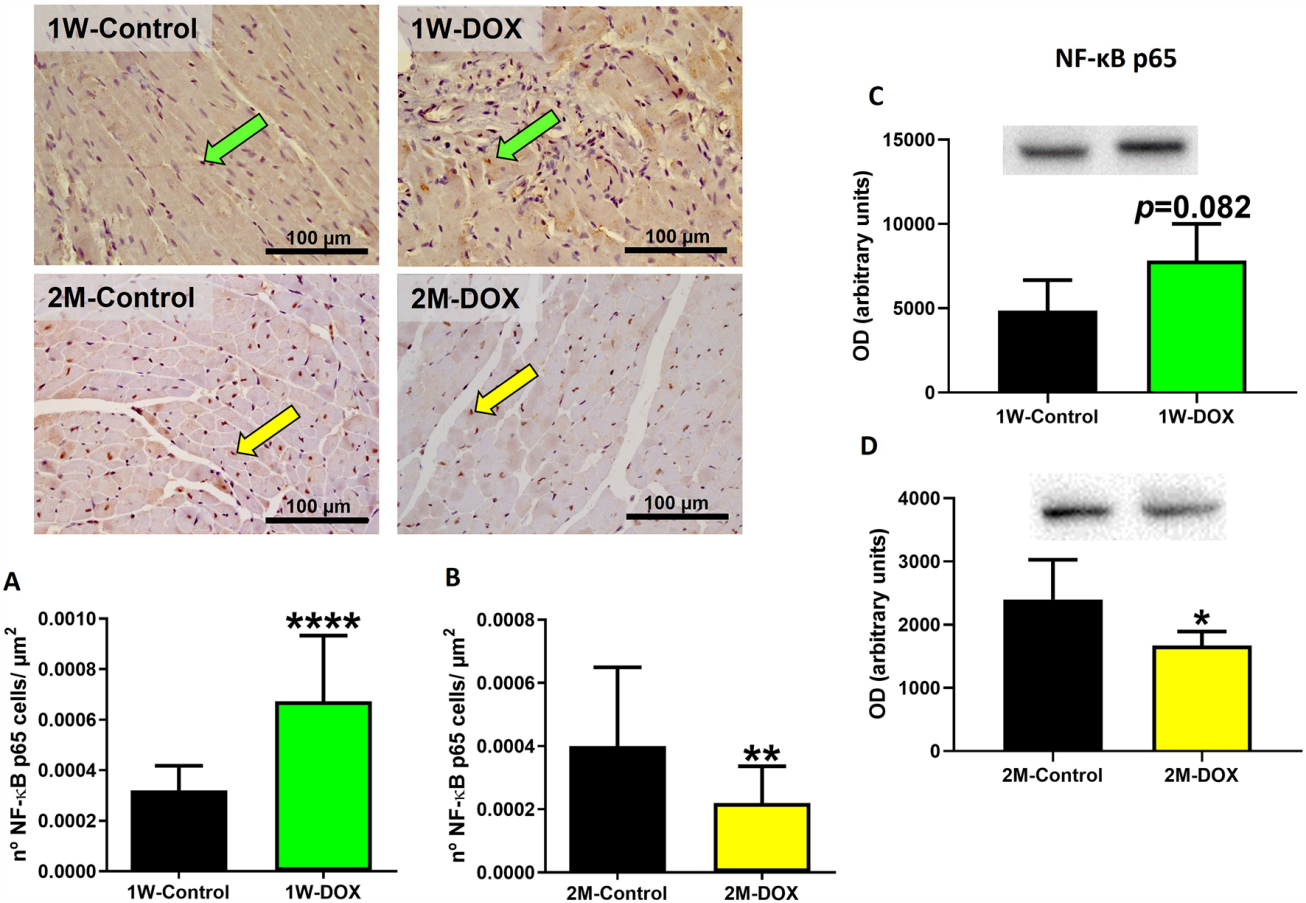
Representative photomicrographs of the immunohistochemistry determination of nuclear factor kappa B (NF-κB) in the cardiomyocytes cells from DOX-treated elderly mice sacrificed 1 week after the last administration (1W-DOX) and control mice (1W-Control), and DOX-treated elderly mice sacrificed 2 months after the last administration (2M-DOX) and respective controls (2M-Control). **A**, **B** The number of cells staining as positive, indicated by arrows for the activated NF-κB of the heart of DOX-treated and control groups, **A** 1W-Control and 1W-DOX; and **B** 2M-Control and 2M-DOX. The results were expressed according to the number of positive cells per area (µm^2^) as mean ± SD. Results were obtained from three animals from each treatment group (six random fields per *n*). Statistical comparisons were made using the Mann–Whitney test: **p < 0.01, ****p < 0.001,  DOX *vs*. control. Scale bar = 100 µm. Images were taken at 40 × . **C**, **D** NF-κB p65 (60 kDa) expression in the cardiac tissue evaluated by Western blotting, in **C** 1W-Control and 1W-DOX; and [D] 2M-Control and 2M-DOX. Values are expressed as mean ± SD and were obtained from 4 (1W) or 5-6 (2M) animals from each treatment group. Statistical comparisons were made using the unpaired *t *test: *p < 0.05, DOX vs. control. OD: optic density. Protein loading on the Western blot was confirmed by the Ponceau S staining (Figure S4)

Immunohistochemistry evaluation revealed that the 1W-DOX group showed positive cytoplasmic and nuclear expressions of NF-κB p65 (brown staining) when compared with the 1W-Control group (Fig. 6A). In the 1W-DOX group, a tendency to increase the NF-κB p65 expression (*p* = 0.082) was observed when compared with the 1W-Control group (Fig. 6C). In the 2M-DOX group, a significant decrease in the NF-κB p65 expression was observed compared to the 2M-Control group (Fig. 6D).

### The 1W-DOX group had a decrease in p62 and LC3-I expression while the 2M-DOX group showed a decrease in LC3-II and LC3-I expression

**Fig. 7:**
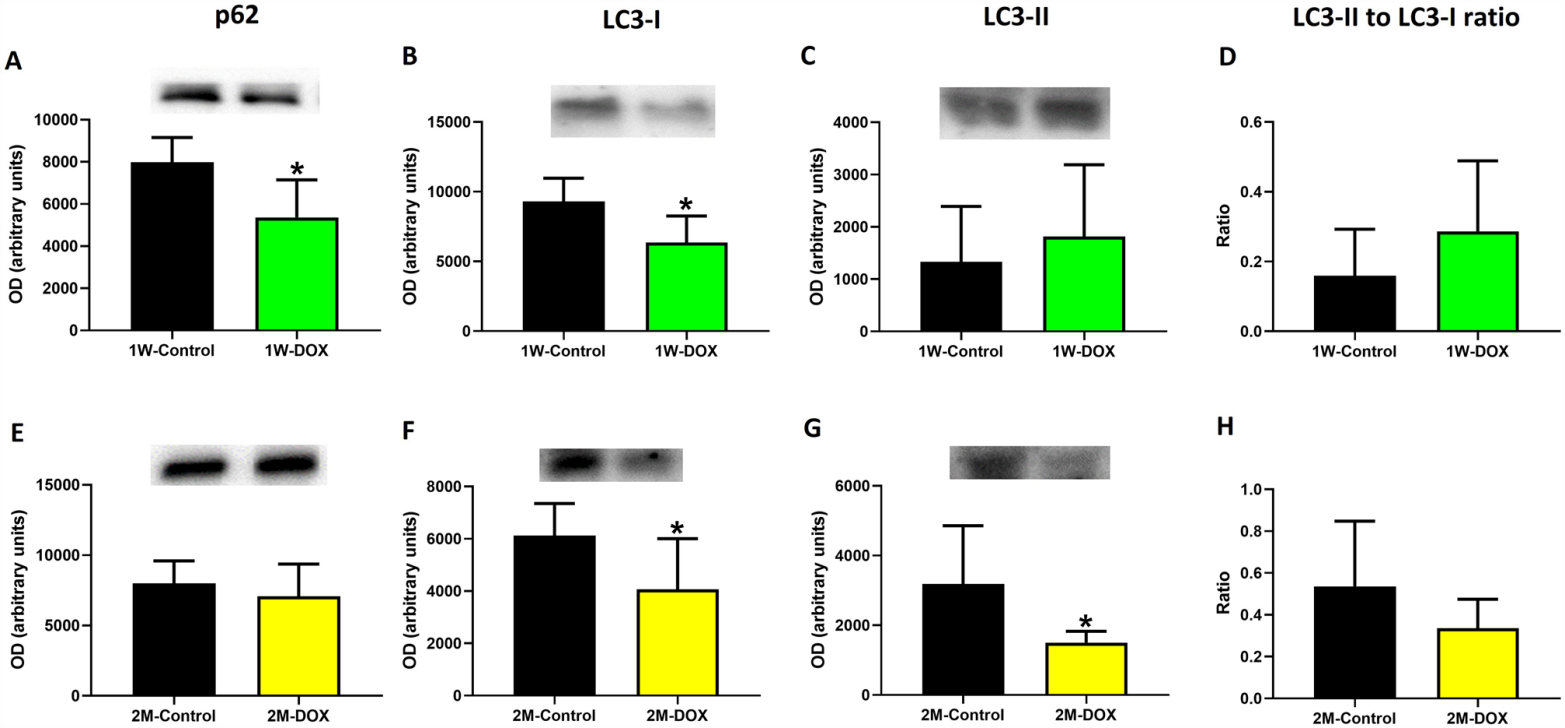
**A**, **E** p62 (62 kDa), **B**, **F** microtubule-associated protein 1A/1B-light chain 3 (LC3)-I (18 kDa), **C**, **G** LC3-II (16 kDa) expression evaluated by Western blotting and the ratio of LC3B-II to LC3B-I in the cardiac tissue, in **A**, **B** elderly mice sacrificed 1 week after the last administration of DOX (1W-DOX) and control mice (1W-Control); and **C**, **D** elderly mice sacrificed 2 months after the last administration of DOX (2M-DOX) and respective controls (2M-Control). Values are expressed as mean ± SD and were obtained from 4-5 (1W) or 5-7 (2M) animals from each treatment group. Statistical comparisons were made using the unpaired *t *test: *p < 0.05, DOX *vs*. control. OD: optic density. Protein loading was confirmed by the Ponceau S staining (Figure S5)

The 1W-DOX group showed a decrease in p62 and LC3-I expression after DOX treatment when compared with the 1W-Control group (Fig. 7A, B), while no changes in LC3-II expression and ratio LC3-II to LC3-I were observed (Fig. 7C, D). The 2M-DOX group showed no significant differences in p62 expression (Fig. 7E) although decreased LC3-I and LC3-II expressions were seen, compared to the 2M-Control group (Fig. 7F, G).

### The 2M-DOX group showed a significant increase in Bax expression

**Fig. 8:**
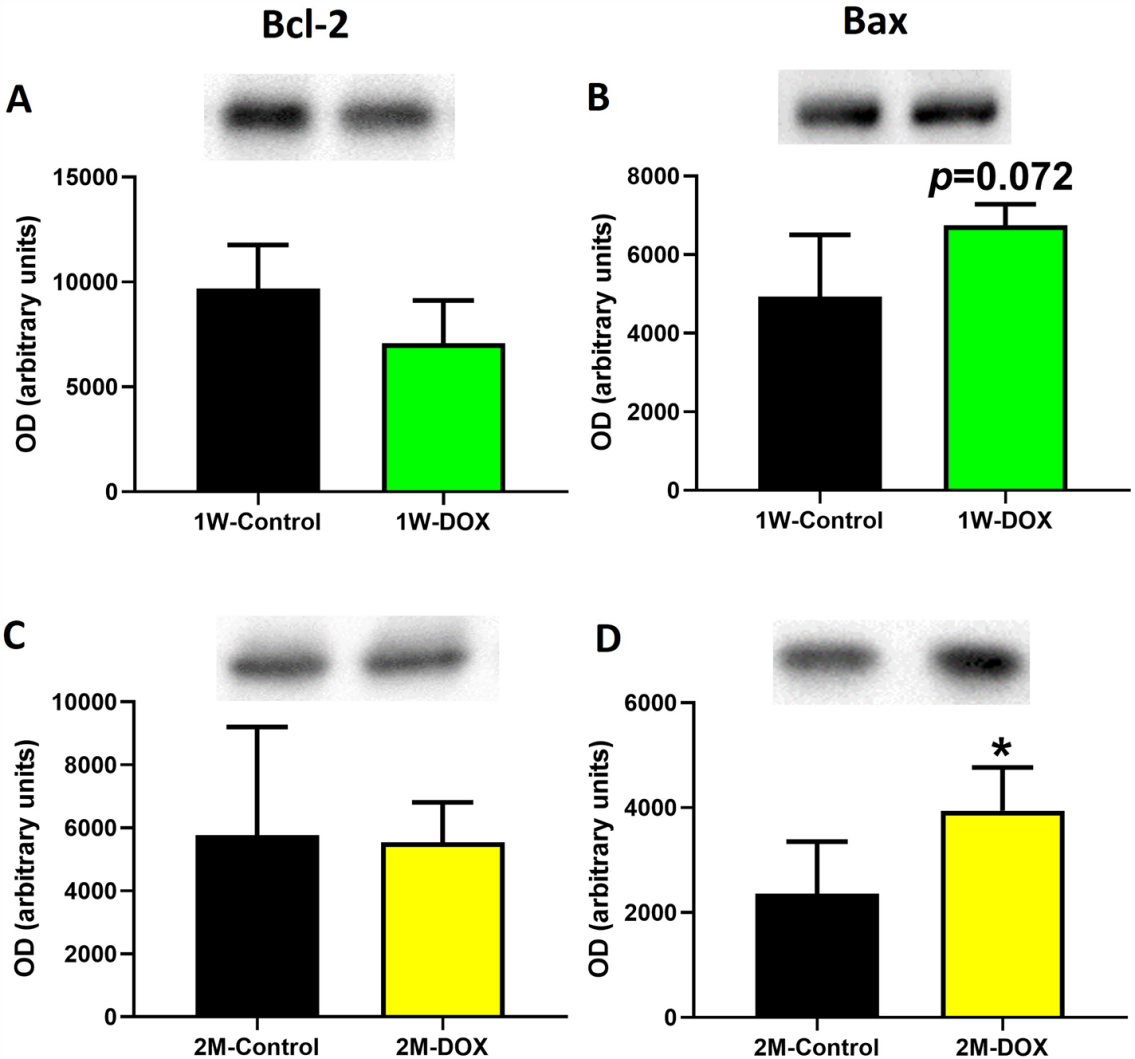
**A**, **C** B-cell lymphoma 2 (Bcl-2) (26 kDa) and **B**, **D** B-cell lymphoma-2-associated X (Bax) (21 kDa) expression in the cardiac tissue evaluated by Western blotting, in **A**, **B** elderly mice sacrificed 1 week after the last administration of DOX (1W-DOX) and control mice (1W-Control); and **C**, **D** in elderly mice sacrificed 2 months after the last administration of DOX (2M-DOX) and respective controls (2M-Control). Values are expressed as mean ± SD and were obtained from 4 (1W) or 5-6 (2M) animals from each treatment group. Statistical comparisons were made using the unpaired *t *test: *p < 0.05, DOX *vs*. control. OD: optic density. Protein loading was confirmed by the Ponceau S staining (Figure S5)

The 1W-DOX group showed a tendency for increased Bax expression in cardiac tissue (*p* = 0.072) after DOX treatment when compared with the 1W-Control group (Fig. 8B), while no changes in B-cell lymphoma 2 (Bcl-2) expression were observed (Fig. 8A). The 2M-DOX group showed no significant differences in Bcl-2 expression (Fig. 8C) although significantly increased Bax expression was seen, as compared with the 2M-Control group (Fig. 8D).

## Discussion

The present work, carried out in elderly mice, had the following the major cardiac findings: (1) DOX treatment resulted in cardiac tissue damage and a significant increase in fibrotic tissue compared to control in both groups, sacrificed 1 week (1W) and 2 months (2M) after treatment ended; (2) iNOS expression increased in the 1W-DOX group, while in the 2M-DOX group, an increase in glutathione peroxidase expression and a decrease of Nrf2 and carbonylated proteins expression were seen; (3) in the 1W-DOX group, a tendency for an increased Bax expression in the cardiac tissue was observed, while in the 2M-DOX group, a significant increase in Bax expression was seen; (4) the 1W-DOX group had a higher number of NF-κB p65 immunopositive cells and a tendency for an increased NF-κB p65 expression in the cardiac tissue, while in the 2M-DOX group, a lower number of NF-κB p65 immunopositive cells and correspondent significant decrease in NF-κB p65 expression were observed; (5) in the 1W-DOX group, a significant increase of IL-33 and a tendency to increase IL-6 expression, and a significant decreased IL-1β and p38 MAPK, p62 and LC3-I expression were observed; in the 2M-DOX group, a tendency for a decrease of myeloperoxidase, LC3-I, and LC3-II expression was detected.

One of the most common hallmarks of DOX cardiotoxicity is the structural damage inflicted and, in our paradigm, using elderly animals, DOX treatment resulted in extensive cardiac tissue damage even in a low clinical cumulative dose. Similar results were reported in our previous work (Reis-Mendes et al. 2021b) and by other authors (Berthiaume et al. 2005; Huang et al. 2021; Iwasaki and Suzuki 1991; Koti et al. 2013; Oliveira et al. 2004; Papadopoulou et al. 1999; Shaker et al. 2018; Wu et al. 2019b; Zhao et al. 2018), but never to the best of our knowledge in elderly mice. Most importantly, cardiac damage persisted even 2 months after DOX treatment.

In response to vascular damage or systemic and local inflammation, endothelium transits from a quiescent state to a state called endothelial dysfunction, which in its more severe form leads to deposition of the underlying extracellular matrix including collagen (Dehghani and Panitch 2020). In the present work, we observed that DOX treatment induced a significant increase in collagen deposition in the cardiac tissue of mice sacrificed 1W or 2M after the last administration of DOX (Renu et al. 2018). Collagen deposition can lead to fibrosis and impaired cardiac function. This can occur through various mechanisms, including oxidative stress and inflammation. In this study, a trend toward an increase in the number of NF-κB p65 immunopositive cells and NF-κB p65 expression was also observed in the 1W-DOX elderly group, which we will discuss later.

DOX redox cycling and subsequent induced oxidative stress is the most frequently proposed mechanism to explain the complex pathophysiology of DOX cardiotoxicity (Kong et al. 2022; Singal and Iliskovic 1998). However, in the present work, we saw very few changes in oxidative/ nitrosative stress-related markers: DOX only increased iNOS expression in the 1W-DOX group, while in the 2M-DOX group, only a significant increase in glutathione peroxidase expression was observed. Overexpression of glutathione peroxidase can attenuate DOX-induced contractile and mitochondrial dysfunction in the heart of mice (Xiong et al. 2006), suggesting a late adaptation process in the heart even 2 months after the drug administration. Furthermore, one of the most common oxidative modifications is protein carbonylation, which can confer loss of structural or functional activity to a protein (Cecarini et al. 2007). A significant decrease in carbonylated proteins was observed in the 2M-DOX group, which may be an adaptive response, as seen in our previous study (Reis-Mendes et al. 2021b), suggesting activation of proteasomal degradation of oxidatively modified proteins and stress response (Wong et al. 2008) or other mechanisms that still need to be clarified.

As previously mentioned, oxidative stress has been linked to DOX cardiotoxicity, with Nrf2 being one of the key players in redox homeostasis (Bhagat et al. 2022; Mata and Cadenas 2021). Nrf2 is crucial in the cellular defense system by controlling xenobiotic and oxidative stress conditions, controlling the expression of antioxidants and detoxifying genes (Kumar et al. 2022). It has been reported that the activation of Nrf2/ARE pathways can protect against DOX-induced cardiotoxicity (Zhou et al. 2022). While in the 1W-DOX group, no significant differences in cardiac tissue Nrf2 expression were observed, a significant decrease in Nrf2 expression in the 2M-DOX group occurred. This can be counterintuitive regarding the data seen in glutathione peroxidase expression that increases; nonetheless, our results suggest that the time elapsed after the last administration is decisive in the observed cardiac effects and adaptative processes. Moreover, this result seems to depend on dose and age, as we observed an increase in Nrf2 expression in infant animals, suggesting that these mice are more protected from damage caused by DOX, 1W after the last administration with a higher cumulative dose (Reis-Mendes et al. 2021b). However, in adult animals with a similar cumulative dose, no significant differences in the Nrf2 expression of cardiac tissue were seen (Reis-Mendes et al. 2021b).

In this study, we also evaluated the autophagy process. Several factors and pathways have been referred to be involved in autophagy. The p38 MAPK pathway has been shown to both positively and negatively regulate autophagy (Sui et al. 2014). LC3-I is the cytosolic form of LC3, which is converted to LC3-II upon induction of autophagy and is incorporated into the autophagosome membrane. Therefore, LC3-II is a marker for autophagic activity (Hennig et al. 2021). Through p62's interaction with LC3, a phagophore membrane is recruited and elongated until it encloses the cargo-p62 complex in an autophagosome. The autophagosome is directed to the lysosome and fuses with the lysosomal membrane for degradation (Berkamp et al. 2021). In the 1W-DOX group, it was observed a significant decrease in p38 MAPK, p62, and LC3-I expression in comparison to the control group. These results suggest a potential impairment in the cellular response to stress and in the autophagic process since LC3-I is required for the elongation of the autophagic membrane. On the other hand, several studies demonstrated that p38 MAPK expression is decreased in cardiac tissues obtained from patients with end-stage heart failure when compared to non-failed heart tissue (Communal et al. 2002; Lemke et al. 2001). In the 2M-DOX group, in addition to a decrease in the LC3-I expression as in the 1W-DOX group, a significant decrease in the LC3-II expression was also observed, suggesting a reduction in autophagic flux, which refers to the complete process of autophagy from initiation to degradation. This decrease in LC3-II expression could occur due to impaired lysosomal function, inhibition of autophagosome-lysosome fusion or could indicate a reduction in the overall number of autophagosomes. Controversial data have been published, with several studies reporting that DOX increases or decreases autophagy in cardiac tissue (Brandão et al. 2022).

It is a known fact that DOX activates the intrinsic apoptotic pathway in cardiac cells, which can cause cell death and contribute to the development of HF (Bhagat et al. 2022). Both DOX-exposed groups had changes in Bax expression: the 2M-DOX group showed a significant increase and in the 1W-DOX group, a tendency was observed. The results suggest that DOX treatment at 9.0 mg/kg can lead to cardiotoxicity in elderly mice through the induction of apoptosis. Our results agree with previous studies that showed a significant increase in Bax protein expression after DOX (Hamza et al. 2016; Saeed et al. 2015).

NF-ĸB is a protein complex that controls the transcription of pro-inflammatory genes (Albensi 2019). Activation of the NF-κB pathway is associated with the aging process and cardiovascular morbidity (Smykiewicz et al. 2018). Moreover, iNOS is one of the target genes for NF-κB activation, and it can modulate NF-κB activity by inhibiting or enhancing downstream pathways (Katsuyama et al. 1998). DOX is a strong iNOS inducer (Aldieri et al. 2002; Lind et al. 1997), it favors the degradation of the IkBα inhibitory complex, and allows the NF-κB factor to translocate to the nucleus and activate the transcription of the iNOS gene (Riganti et al. 2008). In our work, a tendency to increase the number of NF-κB p65 immunopositive cells and its expression in the 1W-DOX group was seen. We also observed an increase in iNOS expression in the 1W-DOX group, which suggests that the increase in iNOS expression exacerbates inflammation and cardiac damage. Other authors showed that DOX leads to inflammation through the NF-κB activation pathway, both in vivo (Benzer et al. 2018; Hamza et al. 2016; Mantawy et al. 2014; Saeed et al. 2015) and in vitro (Guo et al. 2013; Hamza et al. 2016; Wang et al. 2002) studies. DOX activates several inflammatory pathways in cardiac cells, including NF-κB and inflammasome pathways (Bhagat et al. 2022), which leads to the production and release of pro-inflammatory cytokines and chemokines, causing further cardiac cell damage and exacerbating the development of cardiotoxicity (Bhagat et al. 2022; Murphy et al. 2020). Moreover, in the present study, in the 2M-DOX group, a decrease in the number of NF-κB p65 immunopositive cells and NF-κB p65 expression was observed with no changes in iNOS. The 2M-DOX group also showed a trend toward decreased myeloperoxidase expression in comparison to the 2M-Control group. These results suggest that the 2M-DOX group had a decrease in the acute inflammatory response.

Inflammaging, characterized by increased expression of pro-inflammatory cytokines, IL-1, IL-6, and TNF-α, was demonstrated in elderly people with cardiovascular diseases (Smykiewicz et al. 2018). In the 1W-DOX group, significantly decreased expression of IL-1β was observed. The IL-1β suppression by DOX can have detrimental effects on the heart, once IL-1β is involved in various physiological processes in this organ, including the regulation of cardiac function, inflammation, and tissue repair (Szekely and Arbel 2018). The regulation of IL-1 in the heart varied in CD-1 mice of different age groups (infant, adult, and elderly) treated with DOX at a similar cumulative dose and at the same time of sacrifice (1W). In the case of elderly mice treated with DOX, the decrease in IL-1 expression seems to be attributed to age-related changes in the immune system and altered inflammatory responses. TNF-α, IL-6, and IL-1β are pro-inflammatory cytokines involved in DOX-induced cardiotoxicity and are increased in the blood in individuals who have cardiac dysfunction (Miettinen et al. 2008; Murphy et al. 2020; Tamariz and Hare 2010). Still, in the 2M-DOX group, there were no differences in the levels of IL-1β in DOX-treated elderly animals in comparison to the control group.

IL-33 is a member of the IL-1 family, which plays a role in several physiological and pathological processes, including inflammation, immunity, and tissue repair (Molofsky et al. 2015). In our work, in the 1W-DOX group, a significant increase in IL-33 expression in cardiac tissue was observed; however, no significant difference was observed in the 2M-DOX group. Xing et al. showed that serum IL-33 levels in patients with acute myocardial infarction were significantly increased when compared with normal healthy controls and were further enhanced in the heart failure group (Xing et al. 2021). Moreover, IL-33 induces the activation and nuclear translocation of cytosolic NF-κB1 proteins in endothelial cells and cardiac fibroblasts, which results in the production and release of IL-6 (Pinto et al. 2018). Actually, in our work, the 1W-DOX group had a trend toward increased IL-6 expression in cardiac tissue as compared with controls, suggesting that the DOX treatment may lead to damage to the heart muscle cells and impair cardiac function. DOX was reported to cause a significant increase in IL-6 mRNA levels in the hearts of rats (Lou et al. 2004) and an increased IL-6 protein expression in the hearts of mice (Nozaki et al. 2004). IL-6 promotes an inflammatory response, contributing to cardiac hypertrophy when remains chronically elevated (Terrell et al. 2006). In the 2M-DOX group, neither IL-33 nor IL-6 expression in DOX-treated elderly animals was changed. The difference between the two DOX groups (2M-DOX and 1W-DOX) suggests that 2 months after the last administration, there seems to be a meaningful decrease in inflammatory response elicited by DOX treatment.

## Conclusion

To the best of our knowledge, this is the first time that a study has been performed in aged mice treated with a clinically relevant cumulative dose of DOX. In the short term (1W after DOX administration), DOX-induced inflammation and apoptosis in the elderly population. The increased trend of iNOS, NF-κB p65, IL-33, and IL-6 and the decrease in IL-1β appear to exacerbate inflammation and cardiac damage in this DOX-treated group. Increased expression of Bax and decreased expression of p38 MAPK, p62, and LC3-I suggest impaired autophagy and increased apoptotic signaling. In the long term (2M after DOX administration), DOX still induces apoptosis but less exuberant inflammation. In the 2M group, it was observed a decrease in protein carbonylation, and less oxidative damage of proteins is seen, which could be due to increased activity of antioxidant enzymes such as glutathione peroxidase. A decrease in myeloperoxidase and no changes in NF-κB expression in cardiac tissue suggest reduced inflammation-induced ROS production. On the other hand, the decrease in LC3-I and LC3-II expression suggests an impairment in autophagy that combined with an increase in apoptotic signaling, could contribute to the development or progression of cardiac dysfunction. These findings emphasize the importance of continuous cardiac monitoring during the post-treatment phase, emphasizing the necessity for sustained vigilance and attentive care.

### Supplementary Information

Below is the link to the electronic supplementary material.

Supplementary file1 (ODT 16879 KB)

## Data Availability

The data presented in this study are available on request from the corresponding authors.

## Abbreviations

Bax: B-cell lymphoma-2-associated X
Bcl-2: B-cell lymphoma 2
COX-2: Cyclooxygenase-2
DOX: Doxorubicin
HF: Heart failure
IL: Interleukin
iNOS: Inducible nitric oxide synthase
i.p.: Intraperitoneal
LC3: Microtubule-associated protein 1A/1B-light chain 3
NF-κB: Nuclear factor κB
Nrf2: Transcription factor nuclear factor erythroid-2-related factor 2
MAPK: Mitogen-activated protein kinase
mtDNA: Mitochondria include mitochondrial DNA
ROS: Reactive oxygen species
SOD2: Superoxide dismutase 2
SD: Standard deviation
TNF-α: Tumor necrosis factor-α
TNFR1: Type 1 tumor necrosis factor receptor
TNFR2: Type 2 tumor necrosis factor receptor
1W: One week
1W-DOX: Group of animals euthanized 1 week after the last administration of 9.0 mg/kg DOX
1W-Control: Control group euthanized 1 week after the last administration of saline
2M: Two months
2M-DOX: Group of animals euthanized 2 months after the last administration of 9.0 mg/kg DOX
2M-Control: Control group euthanized 2 months after the last administration of saline
