# Supplementary material for: The role of inflammation and antioxidant defenses in the cardiotoxicity of doxorubicin in elderly CD-1 male mice
Source: Arch Toxicol. 2023 Sep 7;97(12):3163–77. doi: 10.1007/s00204-023-03586-1 (PMC10567829; doi:10.1007/s00204-023-03586-1)
Supplement: Supplementary file 1 [file 204_2023_3586_MOESM1_ESM.odt]

Supplementary Data 

The role of inflammation and antioxidant defences in the cardiotoxicity of doxorubicin in elderly CD-1 male mice 

Ana Reis-Mendes1,2,, Mariana Ferreira1,2,, José Alberto Duarte3,4, Margarida Duarte-Araújo5,6, Fernando Remião1,2, Félix Carvalho1,2, Emília Sousa7,8, Maria Lourdes Bastos1,2 and Vera Marisa Costa1,2,

1Associate Laboratory i4HB - Institute for Health and Bioeconomy, Laboratory of Toxicology, Department of Biological Sciences, Faculty of Pharmacy, University of Porto, 4050-313 Porto, Portugal; 2UCIBIO - Applied Molecular Biosciences Unit, REQUIMTE, Laboratory of Toxicology, Department of Biological Sciences, Faculty of Pharmacy, University of Porto, 4050‐313 Porto, Portugal; 3Research Center in Physical Activity, Health and Leisure (CIAFEL), Laboratory for Integrative and Translational Research in Population Health (ITR), Faculty of Sport, University of Porto, 4200‐450 Porto, Portugal; 4TOXRUN – Toxicology Research Unit, University Institute of Health Sciences, CESPU, CRL, 4585-116 Gandra, Portugal; 5LAQV/REQUIMTE, University of Porto, 4050‐313 Porto, Portugal; 6Department of Immuno-Physiology and Pharmacology, Institute of Biomedical Sciences Abel Salazar, University of Porto, 4050‐313 Porto, Portugal; 7Laboratory of Organic and Pharmaceutical Chemistry, Chemistry Department, Faculty of Pharmacy, University of Porto, 4050‐313 Porto, Portugal; 8CIIMAR – Interdisciplinary Centre of Marine and Environmental Research, 4450‐208 Porto, Portugal.

Corresponding authors: 

Ana Reis-Mendes,

E-mail address: afreis.mendes@gmail.com,

Postal address: UCIBIO/REQUIMTE, Laboratório de Toxicologia, Faculdade de Farmácia, Universidade do Porto, Rua de Jorge Viterbo Ferreira, 228, 4050-313, Porto, Portugal.

Vera Marisa Costa,

E-mail address: veramcosta@ff.up.pt,

Postal address: UCIBIO/REQUIMTE, Laboratório de Toxicologia, Faculdade de Farmácia, Universidade do Porto, Rua de Jorge Viterbo Ferreira, 228, 4050-313, Porto, Portugal; Tel.: +351-220-428-599.

Table S1. Supplier, reference, host species, and dilutions for antibody used in Western blot and immunohistochemistry.

| Primary antibody | Supplier, reference | Host species | Dilution for Immunohistochemistry | Dilution for Western Blot |
| --- | --- | --- | --- | --- |
| B-cell lymphoma 2 associated x (Bax) | Abcam (Cambridge, UK), #ab32503 | rabbit, monoclonal | - | 1:1000 |
| B-cell lymphoma 2 (Bcl-2) | Santa Cruz Biotechnology (Heidelberg, Germany), #sc-7382 | Mouse, monoclonal | - | 1:1000 |
| Catalase | Abcam (Cambridge, UK), #ab16731 | rabbit, polyclonal | - | 1:500 |
| Cyclooxygenase-2 (COX-2) | Abcam (Cambridge, UK), #ab15191 | rabbit, polyclonal | - | 1:1000 |
| Dinitrophenyl-KLH | Invitrogen/Life Technologies (Grand Island, NY, USA), #A6430 | rabbit, polyclonal | - | 1:1000 |
| Glutathione peroxidase 1 | Abcam (Cambridge, UK), #ab22604 | rabbit, polyclonal | - | 1:1000 |
| Inducible nitric oxide synthase (iNOS) | Abcam (Cambridge, UK), #ab15323 | rabbit, polyclonal | - | 1:1000 |
| Interleukin (IL)-1β | Thermo Fisher Scientific (Waltham, MA, USA), #P420B | rabbit, polyclonal | - | 1:1000 |
| IL-33 | Abcam (Cambridge, UK), #ab187060 | Rabbit, monoclonal | - | 1:1000 |
| IL-6 | Abcam (Cambridge, UK), #ab208113 | rabbit, polyclonal | - | 1:1000 |
| Microtubule-associated protein 1A/1B-light chain 3 (LC3) | Sigma-Aldrich (St. Louis, MO, USA), #L7543 | rabbit, polyclonal | - | 1:1000 |
| Myeloperoxidase | Abcam (Cambridge, UK), #ab139748 | rabbit, polyclonal | - | 1:500 |
| Nuclear factor κB (NF-κB) p65 | Abcam (Cambridge, UK), #ab16502 | rabbit, polyclonal | 1:50 | 1:1000 |
| Transcription factor nuclear factor erythroid 2-related factor 2 (Nrf2) | Abcam (Cambridge, UK), #ab31163 | rabbit, polyclonal | - | 1:1000 |
| p38 mitogen-activated protein kinase (MAPK) | Cell Signaling Technology through Werfen (Carnaxide, Portugal), #9212 | rabbit, polyclonal | - | 1:1000 |
| p62 | Abcam (Cambridge, UK), #ab56416 | Mouse, monoclonal | - | 1:1000 |
| Superoxide dismutase 2 (SOD2) | Abcam (Cambridge, UK), #ab13534 | rabbit, polyclonal | - | 1:1000 |
| Tumour necrosis factor-α (TNF-α) | Abcam (Cambridge, UK), #ab66579 | rabbit, polyclonal | - | 1:500 |
| Secondary antibody |  |  |  |  |
| anti-rabbit IgG-horseradish peroxidase | Abcam (Cambridge, UK), #ab97051 | Goat, polyclonal | 1:100 | 1:10000 |
| anti-mouse IgG-horseradish peroxidase | Abcam (Cambridge, UK), #ab6728 | Rabbit, polyclonal | - | 1:2000 |

Figure S1. (A, B) Body weight, (C, D) food, and (E, F) water intake in elderly mice sacrificed one week after the last administration of DOX (1W-DOX) (green squares) and control mice (1W-Control) (black circles); and elderly mice sacrificed two months after the last administration of DOX (2M-DOX) (yellow squares) and respective controls (2M-Control) (black circles). Results of body weight are presented in grams (g) and of food intake/day/weight, mL of water intake/day/weight of the animal, as means ± standard deviation (SD), from 5 (1W) or 8-9 (2M) mice per group. Statistical comparisons were made using a two-way ANOVA followed by Sidak’s post hoc test. (G, H) Heart weight-to-brain weight ratio in 1W-DOX and 1W-Control mice; and 2M-DOX and 2M-Control mice. Statistical comparisons were made using the Mann-Whitney test. 

Figure S2. Loading control of Ponceau S staining of  (A, F) Glutathione peroxidase (22 kDa), (B, G) catalase (60 kDa), (C, H) superoxide dismutase 2 (SOD2) (26.6 kDa), (D, I) inducible nitric oxide synthase (iNOS) (131 kDa) and (E, J)  nuclear factor erythroid-2 related factor 2 (Nrf2) (97 kDa)  from mice treated with DOX or respective controls sacrificed (A, B, C, D E) 1 week or (F, G, H, I, J) 2 months after the last administration.

Figure S3. Loading control of Ponceau S staining of  (A, D) interleukin-1 beta (IL-1 beta) (35 kDa), (B, E) Interleukin-6 (IL-6) (23 kDa) and Interleukin-33 (IL-33) (33 kDa), (C, F) Tumor necrosis factor- α (TNF-α) (25 kDa), type 1 TNF receptor (TNFR1) (50 kDa) and type 2 TNF receptor (TNFR2) (75 kDa) from mice treated with DOX or respective controls sacrificed (A, B, C) 1 week or (D, E, F) 2 months after the last administration.

Figure S4. Loading control of Ponceau S staining of (A, E) p38 mitogen-activated protein kinase (MAPK) (40kDa), (B, F) cyclooxygenase 2 (COX2) (75 kDa), (C, G) Myeloperoxidase (63 kDa) and (D, H) NF-κB p65 (60 kDa) from mice treated with DOX or respective controls sacrificed (A, B, C, D) 1 week or (E, F, G, H) 2 months after the last administration.

Figure S5. Loading control of Ponceau S staining of (A, D) p62 (62 kDa), (A, E) microtubule-associated protein 1A/1B-light chain 3 (LC3)-I (18 kDa) and LC3-II (16 kDa), (B, F) B-cell lymphoma 2 (Bcl-2) (26 kDa) and (C, G) B-cell lymphoma 2 associated x (Bax) (21 kDa) from mice treated with DOX or respective controls sacrificed (A, B, C) 1 week or (D, E, F, G) 2 months after the last administration.
